# DNA Methyltransferase 3b Is Dispensable for Mouse Neural Crest Development

**DOI:** 10.1371/journal.pone.0047794

**Published:** 2012-10-18

**Authors:** Bridget T. Jacques-Fricke, Julaine Roffers-Agarwal, Laura S. Gammill

**Affiliations:** 1 Department of Genetics, Cell Biology and Development, University of Minnesota, Minneapolis, Minnesota, United States of America; Texas A&M University, United States of America

## Abstract

The neural crest is a population of multipotent cells that migrates extensively throughout vertebrate embryos to form diverse structures. Mice mutant for the de novo DNA methyltransferase *DNMT3b* exhibit defects in two neural crest derivatives, the craniofacial skeleton and cardiac ventricular septum, suggesting that DNMT3b activity is necessary for neural crest development. Nevertheless, the requirement for DNMT3b specifically in neural crest cells, as opposed to interacting cell types, has not been determined. Using a conditional *DNMT3b* allele crossed to the neural crest cre drivers *Wnt1-cre* and *Sox10-cre*, neural crest *DNMT3b* mutants were generated. In both neural crest-specific and fully *DNMT3b*-mutant embryos, cranial neural crest cells exhibited only subtle migration defects, with increased numbers of dispersed cells trailing organized streams in the head. In spite of this, the resulting cranial ganglia, craniofacial skeleton, and heart developed normally when neural crest cells lacked DNMT3b. This indicates that DNTM3b is not necessary in cranial neural crest cells for their development. We conclude that defects in neural crest derivatives in *DNMT3b* mutant mice reflect a requirement for DNMT3b in lineages such as the branchial arch mesendoderm or the cardiac mesoderm that interact with neural crest cells during formation of these structures.

## Introduction

During early development, cell lineages are determined by the cell type-specific activation of differentiation programs, with concomitant repression of unexpressed developmental pathways. To shut down alternate paths, developmental gene expression is silenced through epigenetics [1], [2]. Epigenetic silencing happens in a step-wise fashion. After fertilization, repressive parental DNA methylation is removed or lost, giving the pluripotent cells of the early embryo the capacity to express genes associated with any cell type as inductive signals are received [3]. As development proceeds, new epigenetic marks, including methylation of histones and DNA, are laid down on promoters to silence gene expression inappropriate for that time and place in the embryo, gradually “locking in” cell fate decisions and restricting developmental potential [2], [4]. Thus, a key factor in understanding development, and tackling developmental pathways gone awry (as in cancer), is to define the mechanisms that impose epigenetic marks in developmental lineages.

The neural crest is a crucial vertebrate developmental cell population. Neural crest cells arise in the dorsal neural tube, which will form the roof plate of the brain and spinal cord; however, unlike their fate-restricted sisters, neural crest cells are multipotent. After they undergo an epithelial to mesenchymal transition, neural crest cells migrate away from the developing central nervous system and form a staggering array of derivatives throughout the embryo: craniofacial bone and cartilage, the cardiac outflow tract, peripheral neurons, and Schwann cells to name a few [5], [6], [7]. Epithelial to mesenchymal transition has been linked to the acquisition of stem cell properties [8], [9] and changes in DNA methylation [10], [11] in cancer cells, but this connection has not been investigated in the neural crest. To evaluate this mechanism, first and foremost the DNA methyltransferase that regulates neural crest cell DNA methylation must be defined, and the critical developmental window for its activity must be determined.

Of the two known de novo DNA methyltransferases, 3a and 3b (DNMT3a and DNMT3b), DNMT3b is the most significant for neural crest development. DNMT3a is undetectable in mouse embryos from E4.5–9.5, is found only ventrally in the nervous system at E10.5, and is not necessary for mouse embryonic development [12], [13], [14]. On the other hand, cells of the early embryo produce high levels of DNMT3b, with abundant localization in neurepithelial cells of the early neural plate and dorsal spinal cord [13], [14], [15]. *DNMT3b* mutant mice die between E13.5 and E16.5 with cardiac ventricular septal defects, rostral neural tube abnormalities, and apparent craniofacial malformations [12], [16]. Consistent with this, DNMT3b-deficient zebrafish embryos exhibit reduced neurogenesis and defects in the precursors of craniofacial structures, the pharyngeal arches [17]. Likewise, humans with *DNMT3b* mutations exhibit the rare autosomal recessive disorder Immunodeficiency, Centromeric instability and Facial anomalies (ICF) Syndrome and have craniofacial defects such as hypotelorism, a shorter nose and a wider nasal bridge [18]. Importantly, mice carrying ICF-like hypomorphic *DNMT3b* mutations survive to term and exhibit craniofacial anomalies that resemble human patients [16]. Because *DNMT3b* is upregulated as a consequence of neural crest induction [19], and the craniofacial skeleton and upper cardiac ventricular septum are neural crest derivatives, altogether these results strongly suggest that DNMT3b is necessary for neural crest development.

In spite of the evidence that DNMT3b plays a role in the neural crest, the tissue-specific requirement for DNMT3b in neural crest cells remains to be determined. Neural crest genes are expressed early and at higher levels when *DNMT3b* is knocked down in neurepithelium differentiated from human embryonic stem (hES) cells, suggesting that DNMT3b negatively regulates the neural crest gene expression program [20]. However, it is not known whether DNMT3b is required in neural crest cells, or in neighboring, interacting cells during subsequent developmental events. To understand the importance of DNMT3b activity in neural crest cells during their migration and differentiation, it is necessary to evaluate *DNMT3b* mutant neural crest cells in the context of a non-mutant developing embryo.

In this study, we analyze neural crest development in mice with neural crest conditional deletions of *DNMT3b*. We first show that neural crest cells express DNMT3b. Then, after defining the onset of cre recombinase expression in neural crest cells in *Wnt1-cre* [21] and *Sox10-cre* [22] transgenic embryos using the *ROSA26-lacZ* cre reporter (*R26R*) line [23], we use these cre lines to delete *DNMT3b* in neural crest cells. Surprisingly, *DNMT3b* mutant neural crest cells migrate nearly normally and undergo normal differentiation and morphogenesis into craniofacial and cardiac structures. We conclude that DNMT3b is dispensable in mouse neural crest cells, and that defects in neural crest-derived structures in *DNMT3b* mutant embryos [12], [16] are due to a requirement for DNMT3b in neighboring cell types.

## Materials and Methods

### Ethics Statement

This study was carried out in strict accordance with the recommendations in the Guide for the Care and Use of Laboratory Animals of the National Institutes of Health. The animal protocol was approved by the Institutional Animal Care and Use Committee of the University of Minnesota (Protocol Number 0909A72245).

### Animals

*DDX4-cre* [24], *Wnt1-cre* [21], and *Rosa26-lacZ* reporter [23] mice were purchased from Jackson Laboratories (Bar Harbor, ME). *Sox10-cre* transgenic animals [22] were provided by Drs. William Richardson and Nicoletta Tekki-Kessaris, while conditional *DNMT3b* mice [25] were supplied by Dr. Guoping Fan. Mouse embryos were surgically isolated, with day 0.5 being the day of the plug, into phosphate buffered saline (PBS) on ice and fixed as appropriate for the experiment. Embryos were staged according to [26]. Embryos and animals were genotyped by polymerase chain reaction (PCR) as follows:

allele primers

DNMT3b[25]

*DDX-cre, Wnt1-cre, Sox10-cre*5′-CCTGATGGACATGTTCAGGGATCG-3′

5′-TCCATGAGTGAACGAACCTGGTCG-3′

R26R[23]

### In situ Hybridization and Immunofluorescence

Embryos were fixed for 1 to 2 hours at room temperature to overnight at 4°C in 4% paraformaldehyde. Whole mount in situ hybridization with *Sox10* [27] or *Wnt1* (Image Clone 3983987; [28]) and cryostat sectioning were performed as previously described [29]. Immunofluorescence was completed as described in [30] using 1∶500 anti-DNMT3b (Imgenex; San Diego, CA) or 1∶100 anti-Sox10 (Santa Cruz Biotechnology; Santa Cruz, CA) followed by 1∶250 of the appropriate secondary antibody (Jackson ImmunoResearch; West Grove, PA).

### ß-galactosidase Staining

Embryos were fixed in 2% paraformaldehyde, 0.2% glutaraldehyde, and 0.5% NP-40 in phosphate buffered saline (PBS) for 15–30 minutes at room temperature depending on embryonic age. Embryos were washed in PBS, then incubated in 5 mM potassium ferricyanide (K_3_Fe(CN)_6_), 5 mM potassium ferrocyanide (K_4_Fe(CN)_6_•H_2_O), 2 mM MgCl_2_, and 1 mg/ml X-gal (Gold Biotechnology; St Louis, MO) in PBS in the dark until staining was deemed complete. Embryos were then washed in PBS and post-fixed in 4% paraformaldehyde in PBS for 1 hour at room temperature. Embryos were viewed in whole mount with a Zeiss Discovery V8 stereoscope. Selected embryos were embedded, sectioned with a Leica CM1900 cryostat at 10–20 µm, and imaged on a Zeiss AxioImager A1.

### Histology

E16.5 hearts were surgically isolated and fixed in 4% paraformaldehyde for 24 hours, rinsed for 1 hour in water, and stored in 70% ethanol until sectioned. Following dehydration into ethanol and passage into xylenes, the tissue was infiltrated with paraffin for 3 hours at 59°C. Once the hearts were embedded and sectioned at 6 µm, the paraffin was removed in a 40°C water bath and the sections were hematoxylin and eosin stained using the H&E program on a Tissue-Tek Prisma Autostainer (Sakura; Torrance, CA).

### Skeletal Preparations

Skin and viscera of E16.5 mouse embryos were removed and carcasses were fixed overnight in 95% ethanol. Carcasses were stained in alcian blue solution (0.015% alcian blue, 75% ethanol, 20% acetic acid) overnight then transferred back to 95% ethanol for several hours. After 6 hours in 2% KOH, carcasses were stained with alizarin red solution (0.015% alizarin red, 1% KOH) for several hours. Skeletons were cleared with 1% KOH/20% glycerol for several days, then stored in a 1∶1 mixture of glycerol and 95% ethanol.

## Results

### Neural Crest Cells Express DNMT3b

**Figure 1:**
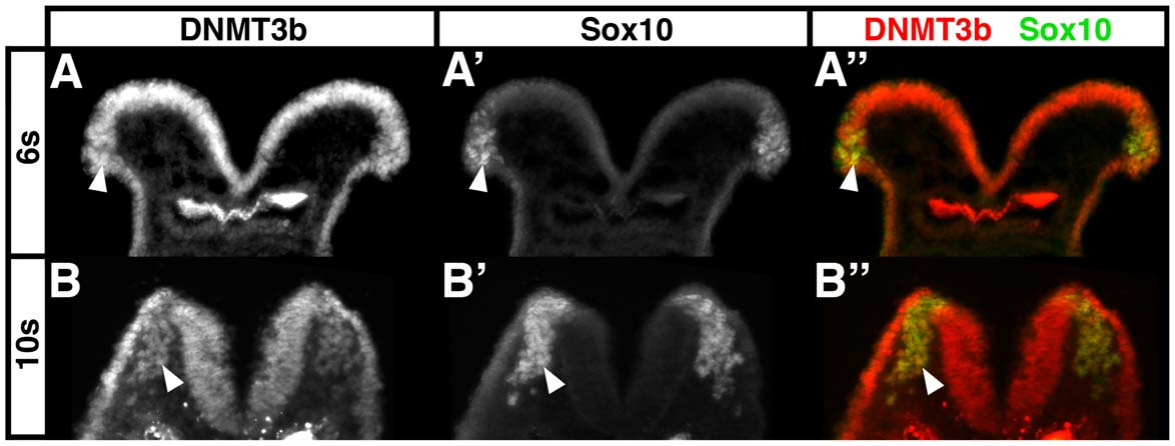
Premigratory and migratory neural crest cells express DNMT3b protein. 6 somite (s; A) and 10 somite (B) wildtype mouse embryos were harvested, 14 µm transverse frozen sections through the midbrain prepared, and DNMT3b (A, B; A’’, B’’ red) and Sox10 (A’, B’; A’’, B’’ green) protein visualized by immunofluorescence. DNMT3b is abundant in the neural plate, including in Sox10-positive neural crest cells in the neural folds (A; arrowheads). Migratory neural crest cells maintain DNMT3b expression (B, arrowheads). The bright staining at the bottom of the images at both stages is in the foregut.

While it is known that the neural plate and dorsal spinal cord express DNMT3b [13], [14], [15], and that DNMT3b is upregulated as a consequence of neural crest induction [19], the DNMT3b expression pattern in neural crest cells has not been determined. By immunofluorescence at 6 somites (E8.0), DNMT3b was abundantly expressed by the neural plate and non-neural ectoderm (Fig. 1A). Costaining with the neural crest marker Sox10 (Fig. 1A’) revealed that the DNMT3b-expressing domain included premigratory neural crest cells in the cranial neural folds (Fig. 1A’’; arrowheads). Similarly at 10 somites (E8.5), once cranial neural crest cells were migratory, the neural plate and non-neural ectoderm continued to exhibit strong DNMT3b immunostaining (Fig. 1B). At this stage, DNMT3b (Fig. 1B), Sox10 (Fig. 1B’) double positive (Fig. 1B’’) migratory neural crest cells (arrowheads) were readily apparent migrating through the DNMT3b-negative head mesenchyme. This indicates that premigratory and migratory neural crest cells express DNMT3b.

### The DNMT3b Conditional Allele Phenocopies the DNMT3b Targeted Mutation

**Figure 2:**
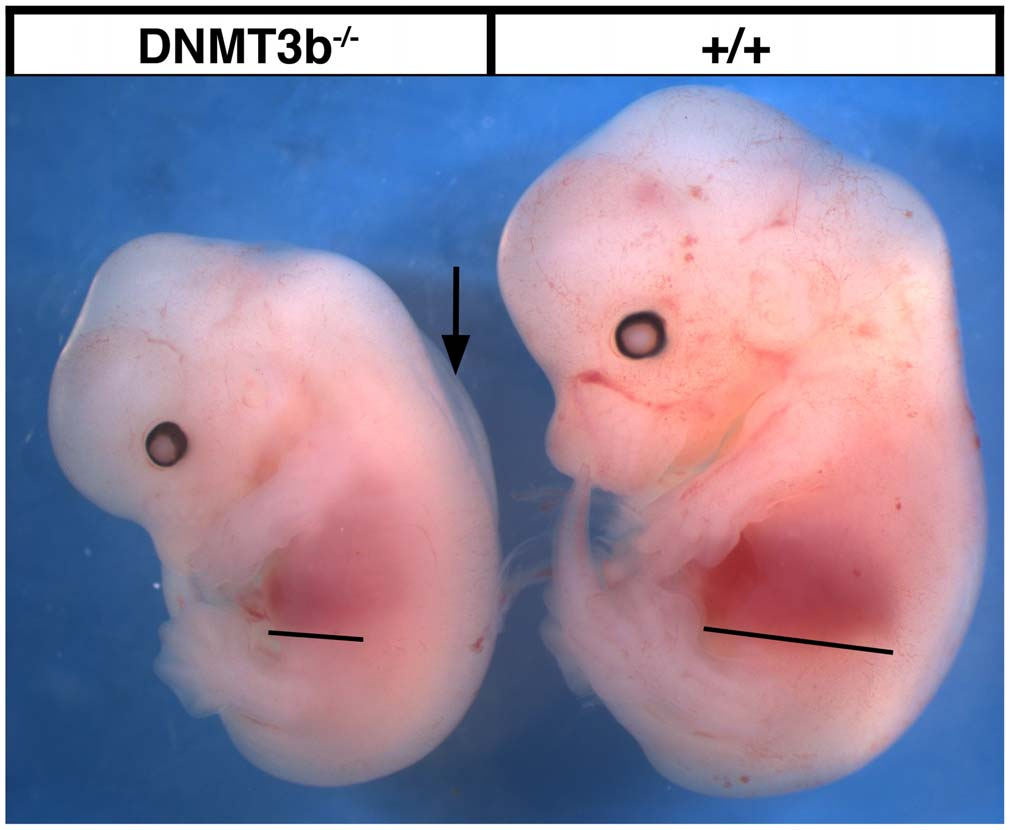
The conditional allele *DNMT3b^−/−^* phenotype. Embryos homozygous for the recombined conditional *DNMT3b* allele (*DNMT3b^−/−^*; left) were compared to wildtype littermates (*+/+*; right) at E13.5. *DNMT3b^−/−^* animals were smaller, had small faint livers (line), and exhibited subcutaneous edema (arrow). These are the same defects exhibited by embryos homozygous for the *DNMT3b* targeted mutation [16].

To define the requirement for DNMT3b specifically in neural crest cells, we took advantage of a line of mice with a conditional *DNMT3b* allele. In these mice, exons 6 through 9 are flanked by loxP sites (floxed, or fl), and when recombined, the essential PC and ENV motifs of the DNMT3b methyltransferase catalytic domain are deleted [25]. Like the targeted *DNMT3b* allele in which the last half of exon 7 through exon 9 are deleted, the conditional *DNMT3b* allele is presumed to be a null [12], [25]. As the conditional allele is nevertheless a distinct mutation, to validate its utility for studying embryonic development, we used *DDX4(Vasa)-cre* germline deleter mice [24] to recombine the *loxP* sites and generate *DNMT3b^−/−^* mice. Like the *DNMT3b^−/−^* targeted mutation [16], conditional allele *DNMT3b^−/−^* embryos are smaller than their wildtype littermates, exhibit subcutaneous edema, and have pale, stunted livers at E13.5 (Fig. 2). Moreover, after dozens of crosses, we have never obtained conditional allele *DNMT3b−/−* pups, indicating the deletion is embryonic lethal. Thus, mice with the recombined *DNMT3b* conditional allele have the same developmental defects as those with a *DNMT3b* targeted allele.

### Wnt1-cre Drives Cre Expression in Premigratory Neural Crest Cells, While Sox10-cre is Expressed as Neural Crest Cells Migrate

The *Wnt1-cre* [21] and *Sox10-cre* [22] transgenic mouse lines both drive cre expression in neural crest cells, and can be used to generate neural crest deletions of conditional alleles. The *Wnt1-cre* transgene efficiently marks neural crest cells at all axial levels and is a commonly used cre line [21], [31], [32]. In *Wnt1-cre* animals, trunk premigratory neural crest cells express cre [33], however, the onset of cre expression in cranial neural crest cells has not been determined. While *Wnt1-cre* drives cre expression in the dorsal third of the neural tube including neural crest cells, cre expression in *Sox10-cre* embryos is neural crest specific; however, *Sox10-cre* has only been detected in migratory neural crest cells [33].

**Figure 3:**
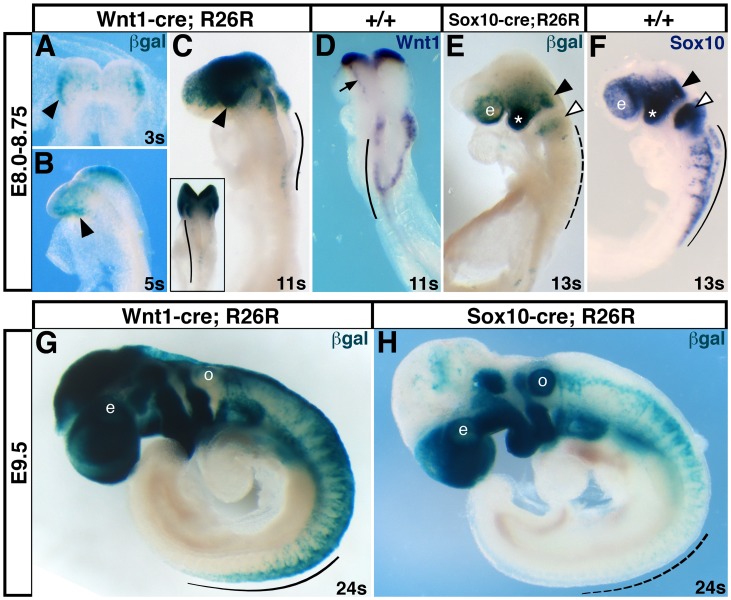
*Wnt1-cre* drives cre expression in premigratory cranial neural crest cells, while *Sox10-cre* is activated during cranial neural crest migration. *Wnt1-cre* and *Sox10-cre* transgenic mice were crossed to mice carrying the *R26R-lacZ* transgene (*R26R*). Embryos were harvested at various time points and stained for ß-galactosidase activity (A–C, E, G, H) or processed by whole mount in situ hybridization for *Wnt1* (D) or *Sox10* (F). At E8.0, *Wnt1-cre* expression was detectable in the anterior neural plate (A, B; arrowheads). By E8.5, robust *Wnt1-cre* expression (C) was apparent in the fore- and midbrain, migratory neural crest cells in the first branchial arch stream (arrowhead), and premigratory neural crest cells in the hindbrain (arc and inset). The *Wnt1* expression pattern (D) for comparison, showing *Wnt1* expression in cranial neural folds (arrow) and hindbrain (arc). At E8.75, *Sox10-cre* activity (E) was strong in the first branchial arch (asterisk), low in neural crest cells entering the first (black arrowhead) and second (white arrowhead) branchial arch streams, and undetectable in trunk premigratory and early migratory neural crest cells (dashed line), although these cells express *Sox10* (F). At E9.5, *Wnt1-cre* (G) marked trunk neural crest cells while they were still in the neural tube (arc), while *Sox10-cre* (H) was not expressed in trunk neural crest cells until migration was well underway (dashed arc). Both transgenes labeled cranial neural crest cells at their destinations. e, eye; o, otic vesicle; s, somite.

**Figure 4:**
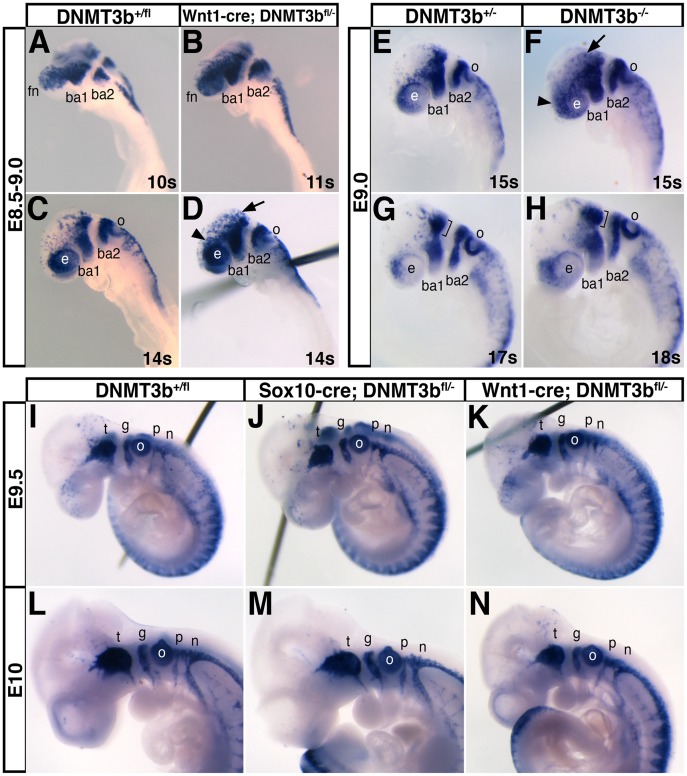
*DNMT3b*-deleted neural crest cells have mild migration defects that recover during cranial gangliogenesis. Migratory neural crest cells and the cranial ganglia they form were visualized by in situ hybridization for *Sox10* at E8.5 (A, B), E9.0 (C–H), E9.5 (I–K) and E10 (L–M). (A–H) E8.5–9.0 embryos that were *DNMT3b^+/fl^* (A, C; wildtype; n = 6); *Wnt1-cre; DNMT3b^fl/−^* (B, D; homozygous deleted in neural crest cells; n = 13), *DNMT3b^+/−^* (E, G; whole embryo heterozygous; n = 6), and *DNMT3b^−/−^* (F; H; whole embryo homozygous deleted; n = 5). Despite normal migration initially (A, B), dispersed *Sox10*-positive cells were apparent dorsally, trailing the organized neural crest streams in the branchial arches (ba; arrows in D, F) and eye (e; arrowheads in D, F) in mutant embryos compared to controls (C, E). Subsequently, neural crest cells coalesce to form the trigeminal ganglia (G, H, brackets). (I–N) At E9.5–10, no difference beyond normal embryonic variation was apparent during cranial gangliogenesis between *DNMT3b^+/fl^* (n = 24), *Sox10-cre; DNMT3b^fl/−^* (n = 6), and *Wnt1-cre; DNMT3b^fl/−^* embryos (n = 13). fn, frontonasal region; g, geniculate; n, nodose; o, otic vesicle; p, petrosal; s, somites; t, trigeminal.

In order to compare the onset and extent of cre recombinase expression in cranial neural crest cells in *Wnt1-cre* and *Sox10-cre* lines side by side, we crossed them to the *R26R* cre-dependent lacZ reporter line [23]. *Wnt1-cre* was active as early as 3 somites (3 s), when low levels of cre expression were detectable in the anterior neural plate (Fig. 3A, arrowhead). This expression increased at 5 s (Fig. 3B, arrowhead), and by 11 s, *Wnt1-cre* expression was apparent in the forebrain, midbrain, and migratory neural crest cells in the first branchial arch stream (Fig. 3C, arrowhead). Premigratory neural crest cells in the hindbrain and trunk also expressed *Wnt1-cre* at this stage (Fig. 3C, arc; inset). The pattern of ß-galactosidase activity in *Wnt1-cre* embryos at 11s included the *Wnt1* expression domain (Fig. 4D) plus migratory neural crest cells, in which *Wnt1-cre* had been expressed when these cells were in the neural folds (arrow).

In contrast, *Sox10-cre* was not expressed until cranial neural crest cells were migratory. Others have detected *Sox10-cre* in migratory neural crest cells as early as ∼10 s [33]. By 13 s, *Sox10-cre* driven cre activity was high in the first branchial arch (asterisk), but low as neural crest cells emerged from the neural folds into the first (black arrowhead) and second (white arrowhead) branchial arch streams (Fig. 3E). Cre activity was not apparent in early migrating neural crest cells in the trunk (dashed arc). This pattern was delayed compared to *Sox10* mRNA, which is strongly expressed in premigratory and migratory neural crest cells (Fig. 3F). Altogether, there appeared to be a lag in *Sox10-cre* expression, such that cre was not strongly expressed until cranial neural crest migration was well underway at a particular axial level (compare ß-galactosidase activity in trunk, second and first branchial arch streams), and cre activity was always lowest dorsally, as neural crest cells emigrated from the neural tube.

At E9.5, a similar pattern continued. As in the head, *Wnt1-cre* marked both premigratory and migratory neural crest cells in the trunk (Fig. 3G, arc), as well as forming neural crest derivatives rostrally. However, because *Wnt1-cre* has a broader expression domain, particularly in the developing brain (see for example [34]), more cell types were labeled, including much of the head (Fig. 3G). In contrast, *Sox10-cre* only labeled trunk neural crest cells once migration was well underway (in other words, the segmental pattern of trunk neural crest migration is only apparent in rostral somites; compare Figs. 3G and H, solid and dashed arcs), and at a distance from the neural tube. However, *Sox10-cre* was a more specific marker of differentiating cranial neural crest derivatives. Only *Sox10-cre* was expressed in the otic placode (Fig. 3H, o). In summary, lineage tracing of neural crest cre drivers indicates that *Wnt1-cre* must be used for examining early events in neural crest development, while *Sox10-cre* expression is more lineage-specific and restricted to neural crest derivatives.

### Despite Subtle Migration Defects, DNMT3b^−/−^ Cranial Neural Crest Cells Form Normal Cranial Ganglia

To determine the requirement for DNMT3b in neural crest cells during their development, we generated *Sox10-cre; DNMT3b^fl/−^* and *Wnt1-cre; DNMT3b^fl/−^* embryos. Neural crest cells were visualized in these embryos by in situ hybridization for *Sox10*, which strongly and specifically marks migratory neural crest cells as well as their derivatives, the cranial ganglia [27]. Because of the late onset of cre expression in *Sox10-cre* lines (Fig. 3; [33]), only *Wnt1-cre; DNMT3b^fl/−^* embryos were examined prior to E9.5.

During migratory stages, DNMT3b loss of function had only mild effects on cranial neural crest migration. At E8.5, neural crest cells were apparent emigrating from the midbrain and hindbrain neural folds in both *DNMT3b^+/fl^* and *Wnt1-cre; DNMT3b^fl/−^* embryos. While initial migration was normal (Fig. 4A, B), as neural crest cells continued ventrally at E9.0, increased numbers of stray *Sox10*-positive cells were dispersed dorsal to branchial arch (ba) streams 1 and 2 (arrow) and next to the eye (arrowhead) in *Wnt1-cre; DNMT3b^fl/−^* embryos (Fig. 4D; 4/4 E9.0 embryos) compared to wildtype (Fig. 4C; 0/5 E9.0 embryos). As this defect was subtle relative to normal embryo-to-embryo variation, we wanted to ensure that *Wnt1-cre* mediated deletion of *DNMT3b* revealed the full extent of this previously uncharacterized migration phenotype. Therefore, we also examined homozygous deleted conditional allele *DNMT3b^−/−^* embryos at these stages. Similarly, at E9.0 in embryos fully mutant for *DNMT3b*, trailing neural crest cells were generally dispersed dorsal to the branchial arches (arrow) and eye (Fig. 4F, arrowhead; 3/3 E9.0 embryos) compared to *DNMT3b^+/−^* embryos (Fig. 4E; 0/3 E9.0 embryos). This indicates that migratory neural crest cells are produced in the absence of DNMT3b, that DNMT3b activity has subtle consequences for neural crest migration, and that *Wnt1-cre; DNMT3b^fl/−^* embryos reflect the neural crest *DNMT3b^−/−^* migratory phenotype.

Despite minor defects in neural crest migration, *DNMT3b^−/−^* cranial neural crest cells successfully coalesced into ganglia. In late E9.0 embryos, the dorsal portion of the ba1 stream began to segregate into the trigeminal ganglia in both *DNMT3b^+/−^* (Fig. 4G) and *DNMT3b^−/−^* embryos (Fig.4H, bracket). By E9.5, the shape, placement, and size of the forming cranial ganglia was equivalent in *DNMT3b^+/fl^* (Fig. 4I), *Sox10-cre; DNMT3b^fl/−^* (Fig. 4J), and *Wnt1-cre; DNMT3b^fl/−^* embryos (Fig. 4K). At E10, the cranial ganglia were comparable in all genotypes (Fig. 4L–N). Therefore, although *DNMT3b* mutant cranial neural crest cells exhibit a subtle migration phenotype, this defect is normalized during cranial gangliogenesis.

### DNMT3b^−/−^ Neural Crest Cells Form Normal Cardiac and Craniofacial Structures

Since mice with a *DNMT3b* targeted deletion or ICF-like point mutations have cardiac and craniofacial defects [16], and because neural crest migration is mildly affected in *DNMT3b* mutants (Fig. 4), we postulated that *DNMT3b* mutant neural crest cells would be impaired in their formation of other neural crest derivatives. To assess this possibility, we harvested *Wnt1-cre; DNMT3b^fl/−^* and *Sox10-cre; DNMT3b^fl/−^* embryos at E16.5 and evaluated cardiac and craniofacial structures.

**Figure 5:**
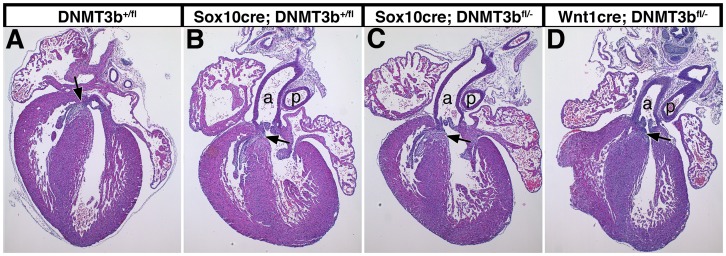
The ventricular septum is intact when cardiac neural crest cells lack functional DNMT3b. E16.5 embryonic hearts from *DNMT3b^+/fl^* (wildtype; A, n = 4), *DNMT3b^fl/−^* or *Sox10-cre; DNMT3b^+/fl^* (heterozygous in neural crest cells; B, n = 8), and either *Sox10-cre; DNMT3b^fl/−^* (C, n = 8) or *Wnt1-cre; DNMT3b^fl/−^* (homozygous deleted in neural crest cells; D, n = 6) were dissected, paraffin sectioned, and hematoxylin and eosin stained. The neural crest-derived upper, membranous ventricular septum (arrows) formed normally in all genotypes. The aorta (a) and pulmonary artery (p) were also correctly attached and arranged (the aorta and pulmonary artery are not apparent in A due to a tilted plane of section).

Contribution of *DNMT3b* mutant neural crest cells to the heart did not affect cardiac development. Cardiac neural crest cells form the aorticopulmonary septum that separates the aorta and pulmonary artery, as well as the membranous portion of the ventricular septum [35]. Aberrant cardiac neural crest development therefore results in conotruncal heart malformations [36], like the upper ventricular septal defects observed in *DNMT3b* mutant embryos [16]. Nevertheless, hematoxylin and eosin stained sections of E16.5 hearts revealed completely enclosed ventricles with no gaps in the ventricular septum in wildtype (Fig. 5A), heterozygous (Fig. 5B) and neural crest mutant animals (Fig. 5C, D, arrowheads). In addition, the aorta and pulmonary artery were distinct and properly attached to the ventricles (Fig. 5 and not shown). Therefore, a heart that includes *DNMT3b* mutant neural crest cells undergoes proper morphogenesis, and *DNMT3b^−/−^* heart defects [16] are not due to a requirement for DNMT3b in cardiac neural crest cells.

**Figure 6:**
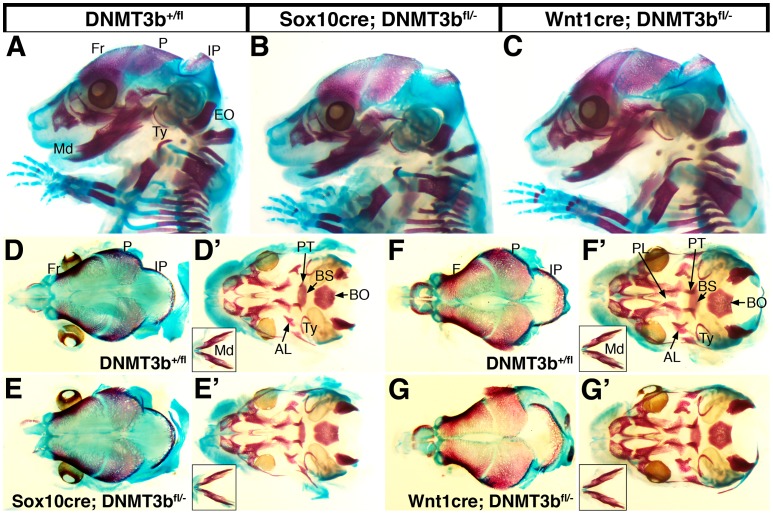
Craniofacial development is normal in neural crest-specific *DNMT3b* mutants. Skeletal preparations of E16.5 embryos from *DNMT3b^+/fl^* (wildtype; A, n = 12) and either *Sox10-cre; DNMT3b^fl/−^* (B, n = 5) or *Wnt1-cre; DNMT3b^fl/−^* (homozygous deleted in neural crest cells; C, n = 7) were alizarin red and alcian blue stained. (A–C) Lateral view; (D–G) skull vault; (D’–G’) skull base, with an inset of the mandible. No defects were apparent in the development, size or placement of cranial skeletal elements. AL, alisphenoid; BO, basioccipital; BS, basisphenoid; EO, exoccipital; Fr, frontal; IP, interparietal; Md, mandible; P, parietal; PT, palatine; Ty, tympanic.

Likewise, neural crest deletion of *DNMT3b* did not affect craniofacial development. *DNMT3b^−/−^* mice die before craniofacial structures form, however, mice with missense mutations equivalent to those in patients with ICF syndrome exhibit delayed skull vault ossification, and shorter, wider frontal bones [16]. To determine if these DNMT3b-dependent abnormalities reflect an essential role for DNMT3b in neural crest cells during craniofacial development, we alizarin red and alcian blue stained skeletal preps of E16.5 mouse embryos. Regardless of genotype, the size, shape, and developmental progress of cranial bones was normal. In a lateral view, all cranial skeletal elements were apparent and exhibited the appropriate length and pattern in wildtype (Fig. 6A) compared to neural crest mutant animals (Fig. 6B, C). Indeed when the skull vault was examined, the frontal, parietal, and interparietal bones were a similar length and width, and ossification was proceeding normally in all embryos (Fig. 6D–G). When viewed from the skull base, the size, shape and position of cranial elements was comparable in control (Fig. 6D’, F’) and neural crest *DNMT3b* mutants (Fig. 6E’, G’), and the mandibles were indistinguishable (insets). Altogether, these data show that DNMT3b is not necessary in neural crest cells for the formation of neural crest derived structures.

## Discussion

*DNMT3b* mutant mice, humans with ICF Syndrome, and mice with ICF-like mutations exhibit craniofacial and cardiac defects [12], [16], [18]. Moreover, neural crest cells express DNMT3b ([19] and Fig. 1). As a result, we postulated that neural crest cells would require DNMT3b for their migration and differentiation. By creating tissue-specific *DNMT3b* knockouts we show, however, that neural crest development proceeds nearly normally in the absence of neural crest DNMT3b function. We therefore conclude that DNMT3b is dispensable for mouse neural crest development.

*DNMT3b* mutant neural crest cells did exhibit subtle migration defects. At E9.0 when most cranial neural crest cells in control embryos had formed coherent streams, there were ectopic *Wnt1-cre; DNMT3b^fl/−^* and *DNMT3b^−/−^* neural crest cells dispersed dorsal to the streams (Fig. 4). This could be because cranial neural crest motility is reduced in the absence of DNMT3b function, so that it takes cells longer to reach their destinations. Alternatively, there could be a defect in collective cell migration [37]. However, these interpretations seem unlikely given that the majority of neural crest cells do organize properly. Another possibility is that the duration of neural crest emigration is extended, resulting in abnormally late migrating, trailing cells that are delayed in their organization into streams. Whatever the cause, embryos recover from this defect, and cranial ganglia (Fig. 4), cardiac neural crest derivatives (Fig. 5), and craniofacial structures (Fig. 6) in neural crest *DNMT3b* mutants are indistinguishable from controls.

The minimal requirement for DNMT3b in neural crest cells is surprising given that animals fully mutant for *DNMT3b* have defects in neural crest derivatives [12], [16]. While DNMT3a compensates for DNMT3b loss of function [12], and the *DNMT3a/3b* neural crest double mutant would likely be more severe, we were specifically interested in determining whether the known phenotypes of *DNMT3b* mutants reflected an essential role for DNMT3b in neural crest cells. Rather, our data suggest that DNMT3b must be required in cell types with which neural crest cells interact during their differentiation, such as the cardiac mesoderm or the branchial arch mesendoderm. It is also conceivable that DNMT3b is required to methylate particular targets during neural crest specification prior to the onset of *Wnt1*-driven cre expression [38], and the consequence of this methylation is not manifest until neural crest cells differentiate during organogenesis. Unfortunately we cannot formally evaluate this possibility as we are not aware of any neural crest cre lines expressed earlier than *Wnt1-cre*, and *DNMT3b^−/−^* embryos die after E13.5 ([16] and our unpublished observations). However, we find this interpretation unlikely for two reasons. First, DNA methylation is normally a late, irreversible event in gene silencing (see below and [1]). Second, neural crest migration was similarly affected in fully mutant *DNMT3b^−/−^* embryos and neural crest mutant *Wnt1-cre; DNMT3b^fl/−^* embryos (Fig. 4). This suggests that DNMT3b is not essential prior to Wnt1-cre mediated recombination. Thus, we favor the interpretation that DNMT3b is required in cell types that interact with neural crest cells during craniofacial and cardiac morphogenesis, and this leads to defects in neural crest derivatives in *DNMT3b* mutants [12], [16].

If neural crest derivatives form normally without DNMT3b, what regulates neural crest epigenetics? A number of studies have demonstrated that gene silencing is a step-wise process, with initial silencing mediated by the formation of repressive chromatin [1]. The consequence of histone modifications, repressive chromatin (or heterochromatin) is flexible and reversible, but is nevertheless sufficient to shut down pluripotent gene expression as development progresses [39]. As a later step, histone methyltransferases and/or histone methylation patterns recruit DNA methyltransferases to heterochromatic sites to bring about stable, long-term repression by DNA methylation [1]. We therefore propose that, in neural crest cells as in other cell types, heterochromatin formation is sufficient to maintain repression of gene expression programs in the absence of DNMT3b, but that over the long-term, DNA methylation will be required for stable repression. Thus we predict that, although they are embryonically normal, with time neural crest lineages in *Sox10-cre; DNMT3b^fl/−^* and *Wnt1-cre; DNMT3b^fl/−^* animals would become epigenetically unstable. In addition, DNMT3a and 3b are functionally overlapping [12], and DNMT3a may provide compensatory DNA methyltransferase activity in the neural crest.

As a dynamic cell type with extended multipotentiality [5], the neural crest likely mobilizes robust heterochromatinization pathways to confer gene silencing. In support of this, the few neural crest heterochromatin regulatory factors that have been identified play crucial roles in neural crest specification, migration, and differentiation [40], [41], [42]. These findings, and the results we report here, emphasize the importance of characterizing additional neural crest heterochromatin regulatory factors.
